# ‘Why would you promote something that is less percent safer than a condom?’: Perspectives on partially effective HIV prevention technologies among key populations in South Africa

**DOI:** 10.1080/17290376.2018.1536561

**Published:** 2018-10-25

**Authors:** Clara Rubincam, Peter A. Newman, Millicent Atujuna, Linda-Gail Bekker

**Affiliations:** a Factor-Inwentash Faculty of Social Work, University of Toronto, Toronto, Ontario, Canada; b Desmond Tutu HIV Foundation, Institute of Infectious Disease, Health Sciences Faculty, University of Cape Town, Cape Town, South Africa

**Keywords:** Biomedical HIV prevention, translational research, qualitative methods

## Abstract

New biomedical prevention technologies (NPTs) for HIV, including oral Pre-Exposure Prophylaxis, and vaginal and rectal microbicides and HIV vaccines in development, may contribute substantially to controlling the HIV epidemic. However, their effectiveness is contingent on product acceptability and adherence. We explored perceptions and understanding of partially effective NPTs with key populations in South African townships. From October 2013 to February 2014, we conducted six focus groups and 18 individual interviews with Xhosa-speaking adolescents (*n* = 14), adult men who have sex with men (MSM) (*n* = 15), and adult heterosexual men (*n* = 9) and women (*n* = 10), and eight key informant (KI) interviews with healthcare workers. Interviews/focus groups were transcribed and reviewed using a thematic approach and framework analysis. Overall, participants and KIs indicated scepticism about NPTs that were not 100% efficacious. Some participants equated not being 100% effective with not being completely safe, and thus not appropriate for dissemination. KIs expressed concerns that promoting partially effective NPTs would encourage substitution of a more effective with a less effective method or encourage risk compensation. Educational and social marketing interventions that address the benefits and appropriate use of partially effective NPTs, including education and support tailored for frontline service providers, are needed to prepare for successful NPT implementation in South Africa.

## Introduction

New prevention technologies (NPTs) for HIV are changing the course of the epidemic. The World Health Organization (2015) recommended oral pre-exposure prophylaxis (PrEP) in November 2015 as a prevention option for all populations at substantial risk of acquiring HIV. PrEP has been approved in several countries (AVAC-PrEPWatch, 2018), including South Africa (Bekker et al., 2016). Other NPTs are at various stages in the development pipeline, including HIV vaccines, and antiretroviral-based vaginal rings and rectal microbicides. While these technologies may exert a substantial long-term impact in controlling HIV epidemics on a population level, evidence suggests they may be only partially effective in preventing HIV acquisition. NPTs may be particularly beneficial in South Africa, with the highest global HIV burden (Interagency Coalition on AIDS and Development [ICAD], 2010; Phillips et al., 2014).

Several clinical trials of NPTs show promising results. The RV144 HIV vaccine trial in Thailand demonstrated 31% efficacy among a moderately at-risk population (Pitisuttithum, Rerks-Ngarm, O'Connell, Kim, & Excler, 2013; Rerks-Ngarm et al., 2009), with new HIV vaccine trials underway, including Southern Africa (Bekker & Gray, 2017; National Institutes of Health, 2017). A dapivarine ring trial (MTN-020), conducted with high risk women in several African countries, demonstrated 27% efficacy overall, with higher efficacy (61%) among women older than 25 years and lower efficacy among younger women (10%) due to differences in adherence (Baeten et al., 2016). Early trials of rectal microbicides among young men who have sex with men (MSM) and transgender women in North America indicate safety and acceptability, with further trials planned (McGowan et al., 2016). In the case of PrEP, 11 randomised controlled trials demonstrated a range of efficacy, with an overall reduction in risk of HIV acquisition of 51% (Fonner et al., 2016). PrEP effectiveness was significantly impacted by adherence, with a 70% reduction in HIV infection in studies with high adherence and no effect in studies with low adherence (Fonner et al., 2016). The broad range of efficacy, both for the same product and across different products, and documented challenges in adherence, indicate the importance of planning for product implementation. This includes evaluating the extent to which key populations understand and accept these technologies.

The concept of ‘partial efficacy’ is complex and may be difficult to explain (Layer, Beckham, Momburi, & Kennedy, 2013; Newman, Duan, Rudy, Roberts, & Swendeman, 2004; Underhill et al., 2016); even health care providers report erroneous understandings (Milford et al., 2016). Yet it is essential for individuals using NPTs to understand that these products are only partially efficacious. This could signify that the product may offer substantial protection to some people in a population, but that not all people may benefit; or that the product may protect against HIV acquisition in a certain percentage of cases overall; or possibly that the product may reduce the severity of HIV infection but not prevent it entirely; or some combination of these different types of efficacy (Bass, 2005). A general understanding of the partial efficacy of NPTs supports the need to combine different methods of prevention (including biomedical, behavioural, and structural) and, in particular, to guard against substitution of a more efficacious method with one that is less efficacious (ICAD, 2010). Comprehension of partial effectiveness may also militate against risk compensation, in which individuals increase their risk behaviours in response to the perceived protection gained from NPTs (Layer et al., 2013; MacPhail, Sayles, Cunningham, & Newman, 2012; Newman et al., 2009a).

Existing studies document challenges with understanding partial efficacy, and highlight the importance of sound communication, education, and community engagement efforts with key populations in planning for product implementation (L'Engle, Lanham, Loolpapit, & Oguma, 2014; Milford et al., 2016; Newman, Duan, Kakinami, & Roberts, 2008). However, few studies to date have investigated in-depth understandings of ‘partial efficacy’ among key populations. Documenting the various meanings and possible misunderstandings of partially efficacious NPTs is important for the design and development of evidence-informed educational and behavioural interventions, as well as providing context for understanding variations in product acceptability and adherence.

Global policy-level recommendations advocate the development of ‘clear communications about the pros and cons’ of NPTs (AIDS Vaccine Advocacy Coalition, 2012); however, there remain a dearth of specific guidelines as to how this can be achieved (Lombardo, 2011). To better inform efforts to communicate risks and benefits of NPTs to potential users and others stakeholders, this study investigates perceptions and understandings of partial effectiveness of NPTs among key populations in South Africa.

## Methods

### Setting

Key populations – adolescents, MSM, and heterosexual adults – were selected and recruited from Masiphumelele and Gugulethu, two informal peri-urban communities located outside Cape Town, South Africa. The communities are characterised by poor living conditions and high rates of poverty and unemployment. Both communities suffer from a generalised HIV epidemic among adult heterosexual men and women (HIV prevalence ∼25%) (Middelkoop et al., 2010), with indications of increasing prevalence among adolescents and MSM (Bekker, Johnson, Wallace, & Hosek, 2015). These communities have been the sites of a number of HIV clinical trials, including of NPTs.

### Participants and data collection

We recruited Xhosa-speaking adolescents (15–17 years old), adult MSM, and adult heterosexual men and women (18+ years). To elicit individual and collective representations of ‘partial effectiveness’, we conducted two focus group discussions (FGDs) and six in-depth interviews (IDIs) within each population. In order to better understand the context, we also conducted IDIs with key informant (KI) policymakers and South African healthcare workers (HCWs).

We developed a semi-structured interview guide to explore awareness and acceptability of NPTs, perceived HIV risk and current methods of prevention, and understanding of product efficacy. Trained Xhosa-speakers conducted FGDs and IDIs. All were digitally recorded, transcribed, and translated into English. To ensure transcription quality, interviews were cross*-*checked with the initial recordings. At the beginning of each FGD and IDI, the interviewers followed a rigorous process of informed consent and explained the different products based on a prescribed script*.* Participants were reimbursed for transportation with a voucher worth 50 Rand (US$5)*.* Ethical approval was obtained from the University of Toronto Research Ethics Board (Ref #: 29273) and the University of Cape Town Research Ethics Committee (Ref #: 008/2013)*.*

### Data analysis

Data were analysed using a thematic approach. In the initial analysis, overarching codes were generated based on the IDI and FGD topic guides. These were modified after re-reading the transcripts. Codes were then shared and discussed among the team before the codebook was finalised. This codebook was used as a guide for analysis. The codebook was imported into NVivo as nodes, which were used to extract text from the transcripts. Extracted text was compiled across all IDIs and FGDs under specific codes and sub-codes. Using framework analysis (Ritchie & Lewis, 2003), these codes were applied to extract meaningful themes. Triangulation of methods (FGDs and IDIs), participants (different key populations and HCWs) and researchers (analysis was implemented by multiple coders in South Africa and Canada), and prolonged engagement with the research sites, support validity.

## Results

From October 2013 to February 2014, we conducted 6 FGDs (5–8 participants per group; *n* = 36), 12 IDIs with key populations, and 8 IDIs with KIs (*N* = 56). Participants were 14 adolescents (8 male, 6 female; 2 FGDs [*n* = 5/FGD], 4 IDIs); 10 heterosexual adult women (1 FGD [*n* = 8], 2 IDIs); 9 heterosexual adult men (1 FGD [*n* = 7], 2 IDIs); and 15 adult MSM (2 FGDs [*n* = 5, *n* = 6], 4 IDIs). The mean age of each population was as follows: adolescents (*x̄* = 15.7, SD = 0.8); adult heterosexual women (*x̄* = 23.2, SD = 5.0); heterosexual men (*x̄* = 25.0, SD = 6.5); and adult MSM (*x̄* = 27.9, SD = 8.7). KI interviews (*n* = 8; 4 male, 4 female) included HCWs and policymakers (*x̄* = 36.7, SD = 9.9).

Interviews and FGDs ranged from 45–80 minutes in duration. Overall themes are presented below along with exemplar quotations for each theme. Quotations are notated with the population and data collection method. Table 1 summarises key findings.

**Table 1.** Key findings and implications for NPTs in South Africa.

Key findings	Population group	Implications
Few participants framed partial effectiveness in a positive light and concurrent usage of NPTs was not well understood	Participants and KIs	The ‘toolkit’ approach to combination HIV prevention needs to be more clearly explained to responsible personnel – doctors, nurses, counsellors and policymakers – charged with advocating and explaining this approach to the general public and key populations
HCWs expressed concerns that NPTs will result in risk compensation	KIs	Communication and supports for HCWs need to explicitly address their concerns about risk compensation behaviour among key populations and collaborate in developing effective prevention messages
Partial effectiveness is interpreted as also signifying uncertain product safety, an unfinished product	Participants and KIs	Safety concerns that may have arisen in response to experiences or knowledge of previous trials should be explicitly addressed; communicators may harness the benefits of mental models or metaphors to convey partially effective products as nevertheless fully safe

NPT: new prevention technology.

### Questioning the ‘Toolkit’ of HIV prevention

In line with the push for ‘biobehavioural’ interventions to promote HIV prevention (GNP+, 2010; Harrison, 2014), facilitators of all FGDs and IDIs framed the concept of ‘partial efficacy’ in a positive light, explaining that these different prevention methods can be used as a ‘toolkit’ for prevention, along with fewer partners, condoms and regular health check-ups, to reduce the risk of HIV acquisition. Nevertheless, this positive framing was picked up and reflected back by only a minority of participants.

An adolescent grasped the need for multiple prevention methods: ‘I would say it assists the condom because the condom is not 100%’ (Male adolescent, FGD). An adult woman related how she would explain partial efficacy to a friend: ‘I would say she needs to assist it because it’s not 100%, assisting it with the condom’ (Heterosexual woman, FGD). Another adolescent echoed this notion of ‘an assist’, saying ‘they [condoms] have to be assisted when you use them you also have to use them together with that thing that you had been using before and use the condom’ (Female adolescent, FGD). One participant used the metaphor of ‘part-timing the condom’ to advocate for multiple methods used in combination: ‘At least since they part-time the condom that there be something even if it’s one that protects them even if it’s not the whole 100%’ (Female adolescent, IDI).

However, the majority of participants viewed the concept of partial efficacy in negative terms and further expressed concerns about the idea of a toolkit for HIV prevention. A nurse KI expressed doubts about promoting multiple methods, suggesting that HCWs should be focusing on condom use promotion instead:

Interviewer (I) So … can you imagine your client … wanting or actually using one of these products?

Participant (P) If you tell them that it is 34% safety … no.

I No? How do you think they would react if you are suggesting it?

P … they would … they would … I don’t know … they would think that we want them to get HIV … because 34% against 99% … of … of … of a condom … 

I Mm.

P Why would you promote something that is less percent safer than a condom? (Nurse, KI)

A similar concern was expressed by an outreach worker, who said, ‘If I find a female saying, “If I am supposed to use condoms … why would I use this? Why use microbicides if I am supposed to use condoms?”’ (Outreach worker, KI). The notion that multiple prevention methods could be used concurrently was also questioned by a MSM participant: ‘But now my question is, when you use the gel do you stop using the condom or do you continue using the condom plus the gel?’ (MSM, IDI).

Some participants specifically indicated that offering alternatives to condoms would lead to lower levels of condom use.

Most of them don’t like condoms … they are using condoms because they have to … but now if you give them something new … they will not use the condom … and use this new thing … of which that you are not sure about … about how the percentage of its safety. (Nurse, KI)

Both participants and KI HCWs expressed uncertainty about the value of partially effective products, along with concerns about a net result of lower prevention effectiveness.

## Concerns about risk compensation

Some KIs expressed the belief that people would engage in increased sexual risk behaviours if they were told that other prevention options existed in addition to condoms. An outreach worker expressed concern that individuals were creating scenarios in which they overestimated the effectiveness of NPTs. In the case of a vaginal ring:

It’s more … some of them say because I have got a ring … so I can sleep around … I can … ‘cause the ring is gonna protect me from getting HIV … from not getting pregnant … from things … . While we are not telling them that, these are the things which they just crack by themselves. (Outreach worker, KI)

Another outreach worker reported concern that people would engage in risk compensation after being told about different prevention methods:

Yah … OK … but … I don’t know … for me if we say … I do have a problem with that because … with people … you know … people are people. They may find out … for example, if we use … like … for example … if we say that … partially effective … so someone will say, ‘let me just take a risk … to see if this could’, … you know … only to find out this … results to HIV infection … . (Outreach worker, KI)

The outreach worker also worried that the concept of partial effectiveness would encourage individuals to gamble with protection: ‘You know! … if we say it’s only partially effective … , “oh let me try and see if … may be it” … you know sometimes people give that hope … “maybe I might not be infected”’ (Outreach worker, KI). Rather than inform people that certain methods conferred only partial protection, some KIs advocated keeping scientific innovations hidden until full protection could be offered:

You know, I would say for me … it’s … I wouldn’t give, I won’t give people like even a hint of … to be excited of … you know … I would say … this is what we are testing … you know … it’s not working now … we are trying … to find out if this could work. (Outreach worker, KI)

Concerns about risk compensation were largely expressed by KIs, who suggested some people would engage in riskier behaviours due to the perceived efficacy of NPTs.

### Conflating partial effectiveness with partial safety

Another dimension of beliefs about NPTs was a conflation of the concept of efficacy with product safety. Some community members and HCWs spoke about various NPTs as both ‘not 100% effective’ and ‘not 100% safe’. A nurse stated: ‘Mhhh. Isn’t this a bit risky because testing it … because you said it is partially safe … so who are you going to test on … on negative patients?’ (Nurse, KI). An outreach worker reiterated this concern, stating, ‘From me it’s – this product you will be using – it’s not 100% safe … or 100% proven that it might protect you … yes it *might* reduce the chances of you getting HIV … ’ (Outreach worker, KI).

Explaining how she would explain these products, an outreach worker suggested it was a matter of time until certain products would be 100% effective:

I am here to tell you about the products that … we are still researching that … until we reach that they can be 100% safe. Yes, they are there … they just came up with the product … ; but they are not yet proven to be 100% safe … . But they are … they might still reduce the chance of getting HIV. (Outreach worker, KI)

When asked how he would explain the concept of ‘partial efficacy’, a heterosexual man suggested it meant that scientific testing was still ongoing:

P I would explain in this way … when I explain to someone who still wants to know about these medicines, according to what I listened to, these medicines are not being used as yet.

I Yes

P It is still being researched to see how they can work

I Yes

P So it’s not yet medicines that have been passed that they are working

I Yes

P It is medicines that if it can be found in research that they are medicines that work, they can be used. (Heterosexual man, IDI)

Related to this idea that partial effectiveness resulted from incomplete scientific investigations was the obverse logic: some participants concluded that HIV prevention programs should wait until the products were fully tested, ensuring, they reasoned, 100% efficacy.

In addition to safety concerns, some participants expressed doubts about the value of products with low to moderate efficacy. A woman expressed discomfort with HIV vaccines, for example: ‘I think I would use it, but then the problem I have, I think the percentage stated here, I think it’s too little; so I’m not sure how it would work because its percentage is too small’ (Heterosexual woman, FGD). Similarly, when asked about various biomedical HIV prevention products, a nurse said: ‘No … it’s … it’s a good product … it’s a good product … but … the percentage * … is* a problem’ (Nurse, KI).

An MSM participant suggested the unknown efficacy of various products created a situation in which one could not trust any method; he would counsel a friend to ‘use both methods at the same time’ because ‘you don’t know which works 100%’ (MSM, IDI).

One result of the anticipated misconstrual of partial efficacy on the part of local communities, and the perception of partial efficacy as indicative of incomplete safety or uncertainty about the product, was that several HCWs reported they would opt not to promote the use of NPTs. An outreach worker indicated it would be preferable to say a product was not effective at all rather than that it was partially effective:

P I don’t know … if maybe … we could say … ‘it’s not working at all.’

I Mm.

P Rather than saying could or partially … you know? (Outreach worker, KI)

Similarly, a doctor explained his reservations about promoting a partially efficacious product: ‘I don’t know how somebody would feel about taking an unproven … or proven … 30% … hmm … ; so … especially knowing long-term effects, etcetera’ (Doctor, KI).

Partial efficacy was thus understood by some participants and KI HCWs as evidence of incomplete testing and of uncertainty, construed as incomplete product efficacy and, therefore, uncertain product safety. A ‘responsible’ approach therefore advocated by several providers was either not to introduce or even discuss the products until greater ‘certainty’ had been attained or to present powerful caveats to the effect that ‘it’s not working at all!’

## Discussion

The success of emerging NPTs in controlling the HIV epidemic is contingent on product acceptability and the capacity of key populations to employ different complementary strategies and technologies to prevent HIV transmission. Although the efficacy of particular NPTs is a crucial factor in product acceptability (Cameron, Newman, Roungprakhon, & Scarpa, 2013; Newman et al., 2006; Newman, Cameron, Roungprakhon, Tepjan, & Scarpa, 2016), efficacy does not ensure uptake and adherence (Newman, Duan, Rudy, & Anton, 2004). Key populations need to value and accept NPTs in the context of their everyday lives for them to be employed correctly and consistently (Atujuna et al., 2018; Newman & Logie, 2010; Newman, Duan, Rudy, Roberts, et al., 2004). Earlier projections suggested biomedical HIV prevention technologies would have lower efficacy (Fauci et al., 2008), but with an outlook toward developing highly efficacious products. Current evidence suggests some NPTs may be unlikely to achieve 90-plus-per cent efficacy. This creates an imperative to integrate product development efforts and roll out with tailored messages and educational interventions that effectively communicate ‘partial efficacy.’ Understanding how key populations and frontline HCWs conceptualise partially efficacious products is crucial for communication and outreach efforts in order to better incorporate and build on their conceptualisations (Newman et al., 2008).

These data suggest that the concept of partial effectiveness is still understood in largely negative terms. Participants raised several key framings which, taken together, suggests work remains for those tasked with promoting the uptake of NPTs.

First, some participants advised that rather than rushing to promote a product with only partial efficacy, scientists and public health officials should wait for a more efficacious product, endorsing the belief that a completely efficacious product just required more time to develop. In this formulation, there is an ‘incompleteness’, something unresolved or unsafe, about a partially efficacious product. This construal may have come from earlier commentary implying NPT efficacy would progress in a linear fashion towards complete efficacy. It also may be supported by the re-emerging primacy of the broader narrative of biomedical responses to HIV to the de-emphasis of behavioural, social and structural responses (Auerbach & Hoppe, 2015; Kippax & Stephenson, 2012). Given the current consensus that achieving a completely efficacious NPT in the near future is unlikely, it is imperative to develop evidence-informed education and social marketing campaigns to shift perceptions around partially effective products. Mental models or metaphors (Chakrapani, Newman, Singhal, Nelson, & Shunmugam, 2013; Newman, Seiden, Roberts, Kakinami, & Duan, 2009b) may assist in communication efforts to explain why a product may be partially effective while highly safe and beneficial.

Second, the conflation of ‘partial effectiveness’ with ‘partial safety’ raises important considerations for future NPT promotion efforts. These data do not reflect whether participants are aware of previous terminated NPT trials, such as one in which the investigational HIV vaccine increased susceptibility to HIV infection for some recipients (Duerr et al., 2012; Reardon, 2013). However, given how news of these events evolves and is interpreted through different communication channels (Essack, Koen, Slack, Lindegger, & Newman, 2012; Newman et al., 2011), it may be important for future outreach and advocacy efforts to address past events that may exacerbate confusion or mistrust of NPTs. Additionally, as the case of PrEP among MSM in the U.S. suggests, the framing of messages to communicate product efficacy (e.g. efficacy ranges versus point estimates) may impart different meanings and influence acceptability (Underhill et al., 2016).

Third, HCWs shared substantial misgivings about the promotion of partially efficacious NPTs; yet, their support is crucial to the dissemination of these products (Milford et al., 2016). Addressing concerns that partially effective NPTs may encourage risk compensation behaviour or that NPTs may specifically discourage condom use is paramount, particularly in the face of competing evidence in regard to actual risk compensation behaviour (e.g. Koester et al., 2017; Westercamp et al., 2017). Tailored training for frontline HCWs is also important, as they may be overwhelmed by another new technology or intervention they are required to become familiar with and explain to their clients, especially when there are caveats for key populations with disproportionately high levels of HIV prevalence.

### Study limitations

Findings from this qualitative investigation may not be generalisable; apropos of qualitative research in general, we aimed to explore in depth the perceptions and emic understandings of key populations in South Africa. However, the results may be transferable to key populations in peri-urban townships in South Africa, which have among the highest HIV prevalence globally. Findings and insights from this study thus merit attention in planning for NPT implementation. Additionally, while most themes emerged across FGDs and IDIs, and participants and KI service providers, the risk compensation theme emerged predominantly from service providers. It may be that such frontline HCWs are better able to foresee and articulate broad community concerns about increases in risk behaviours in contrast to individuals from key populations themselves; the latter also may be motivated to provide socially desirable responses. However, it is notable that the specific themes we identified were not delineated in the initial topic guides; these findings, which tended to cast partially effective products in a negative light, emerged from participants themselves, with evidence from both key populations and KI service providers. In particular, the findings around conflation of partial efficacy and partial safety are, to our knowledge, highly novel, and were not anticipated in our topic guides. Lastly, as with all studies of technologies that have not yet been proven efficacious or licensed, it is possible that acceptability of partially effective NPTs may differ upon actual product introduction.

## Conclusion

Given the primacy of product efficacy to NPT acceptability (Newman et al., 2016; Newman & Logie, 2010), it is vital to assess how key populations conceptualise partially effective products. Rather than merely documenting areas of disagreement or misunderstanding of scientific concepts, our study offers an in-depth exploration of how key populations in South Africa conceptualise and frame the partial effectiveness of NPTs. Results suggest the importance of engagement with community concerns and priorities through social marketing and education campaigns tailored for key populations and frontline service providers. The time prior to the availability of particular NPTs provides a rich opportunity to foster understanding and acceptance of partially effective HIV prevention technologies that can, when used in combination, comprise a formidable toolkit for combination HIV prevention.
